# Catheter Ablation for Frequent Premature Ventricular Contractions or Paroxysmal Supraventricular Tachycardia With Vagal Bradycardia: A New Clinical Application of Superior Vena Cava–Aorta Ganglionated Plexus Modification

**DOI:** 10.1002/clc.70282

**Published:** 2026-03-31

**Authors:** Xinai Meng, Senlin Huang, Liwei He, Tian Liu, Yanjia Chen, Huahua Li, Xingfu Huang

**Affiliations:** ^1^ Department of Cardiology, Nanfang Hospital Southern Medical University Guangzhou China; ^2^ Department of Anesthesiology, Nanfang Hospital Southern Medical University Guangzhou China; ^3^ Department of Cardiology Hezhou People's Hospital Hezhou Guangxi China

**Keywords:** bradyarrhythmia, catheter ablation, paroxysmal supraventricular tachycardia, premature ventricular contractions, superior vena cava–aorta ganglionated plexus, vagus nerve

## Abstract

**Background:**

Frequent premature ventricular contractions (PVCs) or paroxysmal supraventricular tachycardia (PSVT) in patients with bradyarrhythmia is difficult to treat. Cardioneuroablation (CNA) is now considered a promising treatment for vagally mediated bradyarrhythmia.

**Hypothesis:**

Modifying the right atrial superior vena cava–aorta ganglionated plexus (Ao‐SVC GP) improves heart rate and prognosis in vagal bradycardia with tachyarrhythmias.

**Methods:**

We enrolled 110 patients with PVCs or PSVT and vagal bradycardia who underwent catheter ablation. Patients were randomized into the CNA group (*n* = 55) and the control group (*n* = 55). All patients underwent a conventional electrophysiological examination and ablation of PVCs or PSVT. Next, we performed Ao‐SVC GP modification in patients in the CNA group. The primary endpoints included elevation of the basal HR (> 20%) and shortening of the Wenckebach cycle length (WCL) or the AH interval (> 20%).

**Results:**

The immediate success rate of ablation of PVCs and PSVT in both groups was 100%. Compared with those in the control group, patients in the CNA group showed significant improvement in WCL, corrected sinus node recovery time (cSNRT), mean HR, minimum HR, and DC (404.55 ± 71.80 vs. 489.27 ± 85.63; 359.15 ± 52.29 vs. 409.34 ± 59.73; 68.58 ± 8.11 vs. 56.64 ± 4.15; 46.20 ± 4.67 vs. 41.27 ± 3.25; 5.38 (4.23, 6.32) vs. 8.88 (7.17, 9.93), respectively; *p* < 0.05). More importantly, the incidence of syncope in the CNA group was significantly lower (*p* = 0.032 < 0.05), and the improvement in quality of life was greater and more extensive in the CNA group.

**Conclusion:**

The simplified right atrial Ao‐SVC GP ablation effectively treats vagal bradycardia. Additionally, combining it with radiofrequency ablation for concurrent tachyarrhythmia offers a safe and innovative therapy.

## Graphical abstract

This study demonstrates that combined RFCA and right atrial Ao‐SVC GP ablation provides a one‐stop, innovative solution for patients with vagal bradycardia and concomitant tachyarrhythmias, significantly improving symptoms and reducing syncope.

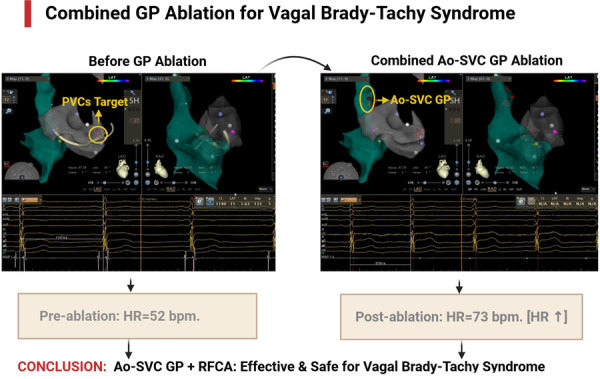

**Abbreviations**
AAD: antiarrhythmic Drug
AO‐SVC GP: superior vena cava–aorta ganglionated plexus
AV: atrial‐ventricle
BCL: basic cycle length
CAN: cardioneuroablation
cSNRT: corrected sinus node recovery time
DC: deceleration force
ECG: electrocardiogram
GP: ganglionated plexus
HR: heart rate
HUTT: head‐up tilt test
LA: left atrial
PSVT: paroxysmal supraventricular tachycardia
PVCs: frequent ventricular premature contractions
QoL: quality of life
RA: right atrial
RFCA: radiofrequency ablation
SA: sinus atrial
SB: sinus bradycardia
SF‐36: the Medical Outcomes Study Short‐Form 36 Health Survey
VVS: vasovagal syncope
WCL: Wenckebach cycle length

## 1 Introduction

Sinus bradycardia (SB) is a common type of bradyarrhythmia and a potential risk factor for cardiovascular events. SB in normal individuals may be asymptomatic or harmless. However, some patients still experience dizziness, chest tightness, reduced activity tolerance, and even syncope [1, 2]. Although relevant studies have confirmed that paroxysmal tachyarrhythmia (such as frequent premature ventricular contractions or paroxysmal supraventricular tachycardia) occurs in a large proportion of people with basal SB [3], there is no effective and safe treatment for this group of people owing to the bradycardia‐inducing adverse effects associated with traditional antiarrhythmic drugs [4, 5].

Cardioneuroablation (CNA) is a promising method for treating symptomatic bradycardia caused by abnormally increased vagal tone [6, 7, 8, 9, 10, 11, 12]. CNA mainly targets the major ganglionated plexi (GPs) of the vagus nerve distributed over the heart [13]. Among these, a group of GPs located on the posterior surface of the right atrium, near the junction of the superior vena cava and the aorta is referred to as the Ao‐SVC GP [14]. As the first site for vagus nerve afferent fibers entering the heart, most vagus nerve fibers that innervate the atria first pass through the Ao‐SVC GP before extending to other atrial ganglia and the atrial surface. Several case reports and small studies have shown that Ao‐SVC GP ablation alone has sustained short‐ and long‐term effects on sinus node function, atrioventricular conduction function, and heart rate improvement [15, 16, 17]. Moreover, radiofrequency ablation (RFCA) can eradicate frequent ventricular premature contractions (PVCs) and paroxysmal supraventricular tachycardia (PSVT). However, to our knowledge, there are currently no clinical studies on the combined use of RFCA and CNA for the treatment of paroxysmal tachycardia with bradyarrhythmia.

Therefore, the aim of this study was to explore the long‐term clinical efficacy of RFCA for PVCs or PSVT combined with right atrial GP ablation for vagal bradycardia through a consolidated radiofrequency ablation procedure. In addition, the efficacy and safety of this simplified GP ablation approach, which is limited to the RA superior vena cava–aorta ganglionated plexus (Ao‐SVC GP), in treating vagal bradycardia were assessed in a long‐term post‐procedure follow‐up study of both groups of patients.

## 2 Methods

The study protocol was approved by the Institutional Ethics Committee of Nanfang Hospital, Southern Medical University (NFEC‐2023‐591), and patients who met all the inclusion criteria and were willing to participate were included after providing written informed consent.

### 2.1 Study Population

We prospectively studied 110 consecutive patients with frequent PVCs or PSVT accompanied by vagal bradycardia who underwent radiofrequency ablation between January 2021 and December 2023 at the Southern Hospital of Southern Medical University. The inclusion criteria for patients with vagal bradycardia were as follows: despite discontinuing antiarrhythmic medications for ≥ 5 half‐lives before the study, the average heart rate on a 24‐h Holter monitor remained < 60 beats per minute, and the heart rate deceleration force (DC) was > 7.5 ms. Patients with a positive atropine test, corrected sinus node recovery time (cSNRT) > 525 ms, or permanent pacemaker implantation for Class I indications were excluded. In addition, patients with a history of structural or organic heart disease, arrhythmias caused by drugs or reversible factors, and any contraindications to cardiac radiofrequency ablation were also excluded. DC is a quantitative indicator of cardiac vagal function, and we included patients whose DC was > 7.5 ms, which was indicative of abnormally increased vagal tone [18, 19]. The atropine test was deemed positive based on an SR < 90 beats/min, junctional arrhythmia, or sinus arrest within 20 min after intravenous administration of 0.03 mg/kg of atropine.

Preoperative data, including heart rate DC and the findings on a 12‐lead electrocardiogram (ECG), 24‐h dynamic monitor (Holter), and echocardiography were evaluated for all patients and used as baseline values. Written informed consent was obtained before study enrollment. Patients were randomly assigned to the CNA group (*n* = 55) or the control group (*n* = 55), with surgery in the control group performed without modification of the Ao‐SVC GP. The randomization schema was created using a computer, and the operators and patients were blinded to the study details.

### 2.2 Electrophysiological Study and Ablation Procedure

All antiarrhythmic drugs were discontinued for at least five half‐lives before surgery. All procedures were performed by electrophysiologists experienced in performing heart denervation procedures, and the same mapping and ablation techniques were applied. All procedures were performed under mild sedation and local anesthesia. A venous fluid circuit was established, and a body surface electrode was attached before the operation. The heart rate, blood oxygen, blood pressure, and respiration were continuously monitored during the operation.

The femoral vein was punctured as usual, and the femoral artery was punctured as needed. A depolarization controllable electrode catheter was placed in the coronary sinus, and a quadrupolar electrode catheter was placed in the right ventricular apex. Intracardiac electrograms were displayed on a multileader recorder. All patients underwent a conventional electrophysiological study before ablation, and the basic cycle length (BCL), Wenckebach cycle length (WCL) and corrected sinus node recovery time were measured.

In the control group, mapping and ablation of PVCs or PSVT were performed as follows. Mapping and ablation of PVCs: Activation mapping and pacing was performed under the guidance of the Carto3 three‐dimensional electrolytic mapping system (Biosense‐Webster) to identify the source of premature ventricular contractions. The target was determined based on the local earliest activation point. RFCA was performed via an ablation catheter (Thermocool SmartTouch [ST] or Thermocool SmartTouch Surround Flow [STSF], Biosense Webster, Inc., Diamond Bar, California), with the ablation parameters set to temperature‐controlled mode, an upper temperature limit of 55°C, a power of 40–45 W, and a local ablation of 40–60 s or AI in the range 400–500. Immediate success was characterized by the disappearance of or only occasional PVCs (≤ 1 bpm) after ablation, less than 10 PVCs during a 30‐min observation period at the end of the ablation procedure, and no ventricular arrhythmias induced by intravenous isoprenaline. Mapping and ablation of PSVT: The mechanism of PSVT was identified via programmed atrial and ventricular stimulation. If the mechanism of PSVT was confirmed to be a bypass, activation mapping was performed to identify the earliest atrial or ventricular activation, and RFCA was performed using the Carto3‐guided system. In the case of dual atrioventricular nodal pathways, RFCA was performed using the Carto3‐guided system and the inferior anatomical localization method. The ablation parameters were set as follows: temperature, 55 ~ 65°C; power, 30 ~ 50 W. Programmed ventricular and atrial stimulation was performed to validate the success of ablation of accessory pathways and dual AV nodal pathways.

In the CNA group, after the ablation of PVCs or PSVT, the ablation catheter was withdrawn to the right atrium. A three‐dimensional Carto3 model of the right atrium and superior vena cava was constructed, and then, the target of Ao‐SVC GP ablation was dissected and ablated [14, 20, 21]. During ablation, the radiofrequency meter was set to temperature control mode, the power was 40 W, the temperature was less than or equal to 43°C, and the cold saline perfusion rate was set to 15 mL/min. The duration of each target discharge was limited by the ablation index (until it reached a value between 350 and 450) (Carto, Biosense Webster). In all patients, the phrenic nerve pathway was localized (by electrical stimulation) before GP ablation to avoid phrenic nerve paralysis. The immediate endpoints of the procedure were defined as an increase in the heart rate of more than 20%, a decrease in the WCL of more than 20%, or a decrease in the AH interval of more than 20%.

After ablation, the BCL, WCL, and cSNRT were measured again to evaluate the ablation effect. Intracardiac electrical signals were filtered in the range of 30 Hz–500 Hz and measured at a scanning speed of 100 mm/s.

### 2.3 Follow‐up

After ablation, all patients were hospitalized for at least 1 day and continued to withhold all antiarrhythmic drugs. The post‐ablation follow‐up visit was conducted in the clinic at 3 and 12 months after ablation and every 6–12 months thereafter. The mean heart rate, minimum heart rate, maximum heart rate, heart rate variability, and heart rate DC were obtained from the 24‐h Holter monitor. Autonomic nervous function is assessed through frequency domain analysis of heart rate variability (HRV), which is used to calculate low‐frequency power (LF), high‐frequency power (HF), and the ratio of LF to HF (LF/HF). Among these, LF reflects the sympathetic nervous system's regulatory role in cardiac rhythm, typically 1170 ± 416 ms². HF reflects the vagus nerve's regulatory effect on cardiac rhythm, typically 975 ± 203 ms². By calculating the LF/HF ratio, the interaction and balance between the sympathetic and vagus nerves can be reflected, with normal values generally ranging from 1.5 to 2.0. Recurrence after PVCs ablation is defined as a PVC burden of 20% or more of the preoperative level at the 3‐month follow‐up [22, 23]. Recurrence after PSVT ablation is defined as the reappearance of PSVT symptoms consistent with the preoperative condition, confirmed by electrocardiogram or electrophysiological examination. In addition, patients were instructed to go to the emergency department if they developed symptoms or to contact medical staff by telephone or online instant messaging.

The Medical Outcomes Study Short‐Form 36 Health Survey (SF‐36) was used to assess quality of life (QoL) before the procedure and 12 months after ablation [7, 24]. The SF‐36 scale includes eight specific areas of quality of life, namely, physical function, limitations due to physical health, physical pain, general health, vitality, and social function, and limitations due to emotional problems and mental health. The score for each subscale ranged from 0 to 100, and the lower the score is, the lower the quality of life.

Each patient conducted a subjective assessment of their symptoms, such as dizziness (syncope or presyncope state), palpitation, and chest tightness, before surgery and 12 months after ablation, and scored them on a scale of 1–10 (mild to severe), with higher scores indicating more severe symptoms. The total score for each patient's symptoms was calculated by adding up the scores of all the individual symptoms.

### 2.4 Statistical Analysis

Continuous variables are expressed as means ± standard deviations, and paired *t*‐tests were used to compare differences between values before and after ablation. Independent sample *t*‐tests or nonparametric tests were used to analyze differences between the two groups, and classified variables are expressed as percentages, which were compared using the chi‐square test or Fisher's exact test. All tests to ascertain significance were bilateral tests, and a probability value < 0.05 indicated significance. All statistical analyses were performed using SPSS version 25.0 (IBM, Armonk, New York).

## 3 Results

### 3.1 Patient Characteristics

One hundred ten patients were included in this study, with 55 patients in the CNA group and 55 patients in the control group. Overall, the patients were 48.50 ± 15.74 years old and mostly male (56.4%). The mean duration of symptoms was 11.13 ± 5.43 months. Dizziness, chest tightness, and palpitation scores > 5 were reported by 65.5%, 79.1%, and 84.5% of the patients, respectively. Additionally, the arrhythmia type, left ventricular ejection fraction, and bi‐atrial sizes were similar in both groups. There were no statistically significant intergroup differences in the mean HR, maximum HR, minimum HR, or degree of heart rate variability on the pre‐ablation ambulatory electrocardiogram. There was no significant intergroup difference in the history of preoperative antiarrhythmic drug administration. The patient's demographic and clinical characteristics are shown in Table 1.

**TABLE 1:** Baseline characteristics of the two groups before the procedure.

	Total patients (*N* = 110)	CNA group (*N* = 55)	Control group (*N* = 55)	*p* value
Age, years	48.50 ± 15.74	46.33 ± 16.95	50.67 ± 14.25	0.148
Male	62 (56.4)	33 (60.0)	29 (52.7)	0.442
BMI, kg/m^2^	23.40 ± 3.10	23.18 ± 3.32	23.62 ± 2.86	0.455
History of symptoms, months	11.13 ± 5.43	10.67 ± 4.97	11.58 ± 5.86	0.382
Hypertension	18 (16.4)	7 (12.7)	11 (20.0)	0.303
Diabetes mellitus	3 (2.7)	1 (1.8)	2 (3.6)	1.000
Preoperative symptom score				
Dizziness ≥ 5	72 (65.5)	38 (69.1)	34 (61.8)	0.423
Chest tightness≥ 5	87 (79.1)	47 (85.5)	40 (72.7)	0.101
Palpitation≥ 5	93 (84.5)	48 (87.3)	45 (81.8)	0.429
Echocardiographic parameters				
LAD, mm	38.54 ± 4.61	37.84 ± 4.55	39.24 ± 4.60	0.112
LVEDD, mm	45.16 ± 4.58	44.64 ± 4.44	45.68 ± 4.69	0.236
RAD, mm	36.05 ± 4.74	35.71 ± 4.06	36.38 ± 5.35	0.459
LVEF, %	63.03 ± 5.42	63.56 ± 5.30	62.49 ± 5.53	0.301
Preoperative Holter				
Type of arrhythmia				0.702
PVCs with SB	50 (45.5)	24 (43.6)	26 (47.3)	
PSVT with SB	60 (54.5)	31 (56.4)	29 (52.7)	
Mean HR, beats/min	54.52 ± 3.10	54.05 ± 3.21	54.98 ± 2.94	0.117
Minimum HR, beats/min	40.52 ± 4.09	40.45 ± 4.67	40.58 ± 3.46	0.871
Maximum HR, beats/min	107.67 ± 13.94	109.04 ± 14.60	106.31 ± 13.24	0.307
PVCs load, %	13.61 ± 4.71	13.17 ± 4.41	14.01 ± 5.03	0.535
HF, ms^2^	535.59 ± 215.11	523.72 ± 220.49	547.47 ± 210.94	0.565
LF/HF	1.27 ± 0.37	1.27 ± 0.46	1.28 ± 0.27	0.170
DC, ms	9.08 (8.26, 10.85)	9.38 (8.20, 11.19)	8.96 (8.27, 10.05)	0.411
AAD application history				
Beta‐blocker	6 (5.5)	2 (3.6)	4 (7.3)	0.679
Propafenone	2 (1.8)	1 (1.8)	1 (1.8)	1.000
Shensong Yangxin Capsule	31 (28.2)	15 (27.3)	16 (29.1)	0.832

*Note:* The values are presented as means ± SDs.

Abbreviations: AAD, antiarrhythmic Drug; AVNRT, atrioventricular nodal reentrant tachycardia; AVRT, atrioventricular reentrant tachycardia; BMI, body mass index; DC, deceleration capacity; HF, high frequency; HR, heart rate; LAD, left atrial diameter; LF, low frequency; LVEDD, left ventricular end‐diastolic diameter; LVEF, left ventricular ejection fraction; PVCs, premature ventricular contractions; PSVT, paroxysmal supraventricular tachycardia; RAD, right atrial diameter; SB, sinus bradycardia.

### 3.2 Procedural Data

The mean procedure times were 105.65 ± 38.25 and 95.33 ± 37.95 min (*p* > 0.05) and the ablation times were 537. 33 ± 160.60 and 421.09 ± 132.34 s in the CNA and control groups (*p* < 0.05), respectively. The mean number of ablation points in patients with Ao‐SVC GP in the CNA group was 5.69 ± 1.40, the mean ablation time was 126.60 ± 36.12 s, and the mean ablation area was 0.72 ± 0.18 cm^2^. The immediate success rate of ablation of PVCs and PSVT in both groups was 100%. There was one case of vascular puncture complication in each of the CNA and control groups, and no other intraprocedural complications were observed.

**FIGURE 1:**
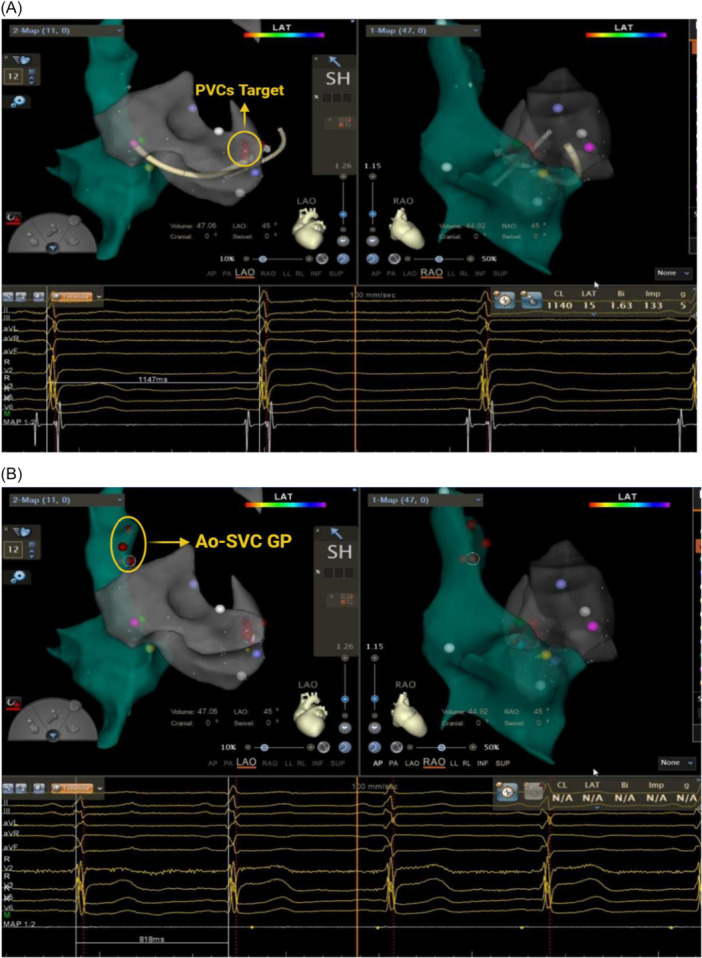
A and B show electrophysiologic examination and labeled target maps in a 52‐year‐old man with PVCs with vagal bradycardia in the CNA group. (A) Schematic of the ablation target for PVCs. The patient's R‐R interval 20 min after PVC ablation was 1147 ms. (B) Ao‐SVC GP electroanatomic labeling and ablation schematic. The R‐R interval was reduced to 818 ms in patients who underwent right atrial cardiac nerve ablation.

The electrophysiologic examination and ablation data are shown in Table 2. The mean immediate HR, BCL, WCL, and cSNRT was significantly improved in the CNA group compared with the control group (77.49 ± 11.12 vs. 59.04 ± 6.14; 751.27 ± 103.01 vs. 1056.53 ± 93.57; 404.55 ± 71.80 vs. 489.27 ± 85.63; 359.15 ± 52.29 vs. 409.34 ± 59.73, respectively; *p* < 0.05). Moreover, in the CNA group, the postoperative values of HR, BCL, WCL, and cSNRT were significantly improved compared to their preoperative values (77.49 ± 11.12 vs. 57.00 ± 8.43; 751.27 ± 103.01 vs. 1106.29 ± 94.00; 404.55 ± 71.80 vs. 509.64 ± 101.56; 359.15 ± 52.29 vs. 411.80 ± 76.30, respectively; *p* < 0.05), indicating that there was a significant improvement in cardiac conduction function in the patients in the CNA group (Table 2, Figure 1). Further analysis revealed that 48 of the 55 patients (87.3%) in the CNA group presented an increase in HR to > 60 beats/min after Ao‐SVC GP ablation, which was significantly greater in these patients than before ablation (80.37 ± 8.58 vs. 57.77 ± 8.65, *p* < 0.01).

**TABLE 2:** Electrophysiological examination and ablation data.

	CNA group (*N* = 55)	Control group (*N* = 55)
PVCs ablation target		
RV outflow tract	12 (50.0)	19 (73.1)
LV outflow tract	8 (33.3)	4 (15.4)
LV papillary muscle	1 (4.2)	2 (7.7)
Valvular ring	3 (12.5)	1 (3.8)
PSVT ablation target		
Slow path	29 (93.5)	14 (48.3)
Left accessory pathway	1 (3.25)	10 (34.5)
Right accessory pathway	1 (3.25)	5 (17.2)
Total procedure time, min	105.65 ± 38.25	95.33 ± 37.95
Total ablation time, seconds	537. 33 ± 160.60	421.09 ± 132.34^a^
Average ablation points of Ao‐SVC GP	5.69 ± 1.40	0
Average ablation time of Ao‐SVC GP, seconds	126.60 ± 36.12	0
Immediate mean HR before operation, beats/min	57.00 ± 8.43	56.96 ± 6.11
Immediate mean HR after operation, beats/min	77.49 ± 11.12^b^	59.04 ± 6.14^a^
Immediate increase in HR, beats/min	20.49 ± 11.57	2.07 ± 5.29^a^
BCL before ablation, ms	1106.29 ± 94.00	1091.98 ± 73.66
BCL after ablation, ms	751.27 ± 103.01^b^	1056.53 ± 93.57^a^
WCL before ablation, ms	509.64 ± 101.56	500.00 ± 87.92
WCL after ablation, ms	404.55 ± 71.80^b^	489.27 ± 85.63^a^
cSNRT before ablation, ms	411.80 ± 76.30	424.41 ± 61.59
cSNRT after ablation, ms	359.15 ± 52.29^b^	409.34 ± 59.73^a^

*Note*: *p* value < 0.05 was considered significant; values are presented as means ± SDs.

Abbreviations: BCL, basic cycle length; cSNRT, corrected sinus node recovery time; LV, left ventricular; RV, right ventricular; WCL, Wenckebach cycle length.

^a^ indicates a significant difference between the CNA group and the control group.

^b^ indicates a significant difference between the groups before and after surgery.

### 3.3 Follow‐up: Clinical Data

In the CNA group, after a mean follow‐up period of 12.82 ± 6.67 (range 4–36) months, two patients (8.3%) experienced PVC recurrence and received treatment with Shensong Yangxin Capsule, and one patient (1.8%) developed sustained sinus tachycardia (HR > 100 beats/min) at 8 months of follow‐up and was treated with metoprolol. Syncope did not recur in 10 patients (83.3%) during the follow‐up period, but two patients (16.7%) developed syncope or presyncope approximately 1 year after the procedure, and the frequency of syncope was significantly lower than that in the preoperative period.

In the control group, after a mean follow‐up of 12.35 ± 5.95 (range 3–34) months, three patients (11.5%) experienced a recurrence of PVCs and were treated with Shensong Yangxin Capsule, and 6 patients (66.7%) had recurrent syncope or presyncope during the follow‐up period. Syncope recurred in three of the six patients within 3 months and in the remaining three patients approximately 1 year after the procedure, and one of the patients underwent pacemaker implantation. No transient ischemic attacks or stroke events were detected in either group during the follow‐up period.

### 3.4 Follow‐up: Electrocardiographic Data

**FIGURE 2:**
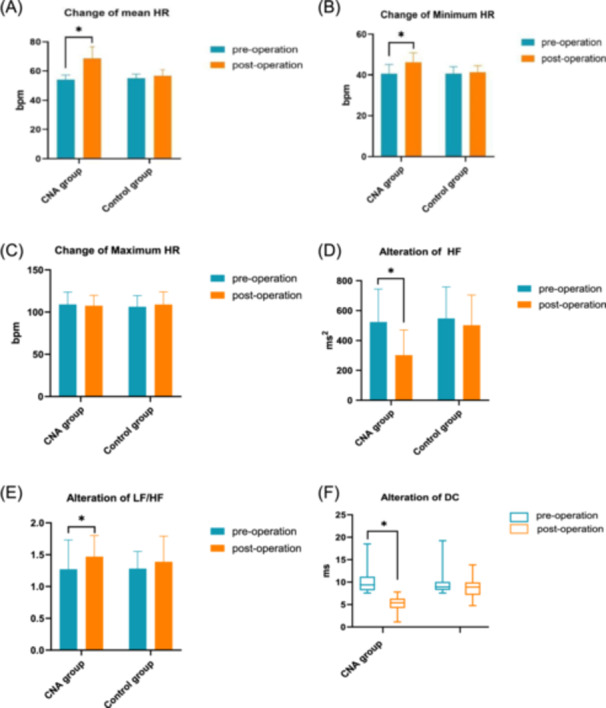
Changes in (A) mean HR, (B) minimal HR, (C) maximum HR, (D) high‐frequency (HF), (E) low‐frequency and high‐frequency (LF‐HF) ratio, and (F) heart rate deceleration force (DC) during the mean follow‐up period in all patients. *p < 0.05 versus before the procedure.

During the mean follow‐up period, the 24‐h mean HR in the CNA group increased from 54.05 ± 3.21 beats/min to 68.58 ± 8.11 beats/min, and the minimum HR increased from 40.45 ± 4.67 to 46.20 ± 4.67 beats/min (*p* < 0.05), whereas the maximum HR did not change significantly from the preoperative period (*p* > 0.05). For changes in heart rate variability, HF and DC decreased significantly after Ao‐SVC GP ablation compared with before (301.98 ± 168.65 vs. 523.72 ± 220.49; 5.38 (4.23, 6.32) vs. 9.38 (8.20, 11.19), respectively; *p* < 0.05), and the LF/HF was elevated, which indicated that the vagal tone was weakened and that the interautonomic tone was more balanced than before the procedure. In contrast, in the control group, there was no significant difference in the mean HR, minimum HR, and maximum HR between the postoperative and preoperative periods (*p* > 0.05), and the HF and DC remained high. The changes in HR and heart rate variability parameters before the procedure and during the follow‐up period are shown in Figure 2.

### 3.5 Follow‐up: Quality of Life

**FIGURE 3:**
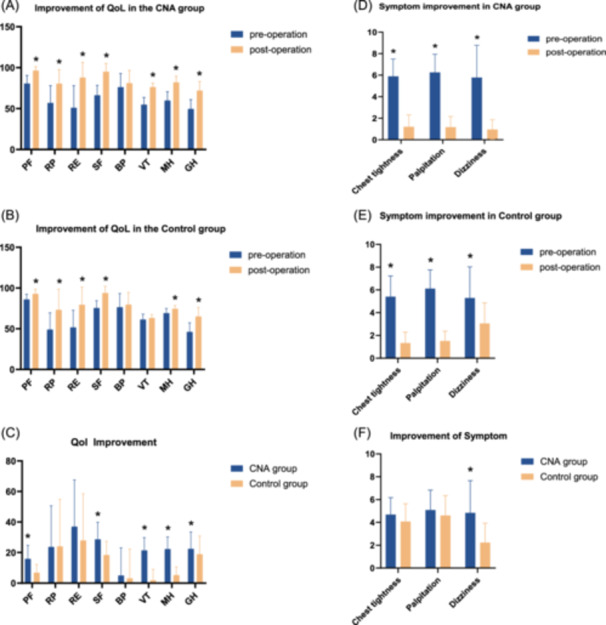
Improvements in quality of life and symptom scores. (A) Improvement in QoL in the CNA group. (B) Improvement in QoL in the control group. (C) Comparison of QoL improvement between the 2 groups. (D) Improvement in symptom scores in the CNA group. (E) Improvement in symptom scores in the control group. (F) Comparison of symptom improvement between the two groups. *p < 0.05 Preoperative versus postoperative or CNA group versus control group.

The quality of life scores assessed before the operation were similar between the two groups, but an analysis of the clinical data during the mean postoperative follow‐up period revealed improvements in seven of the eight subscales of the SF‐36 in the CNA group and in six of the eight subscales of the SF‐36 in the control group (*p* < 0.05 each) (Figure 3A–B). An analysis of all the metrics of the SF‐36 revealed that patients in the CNA group improved more than control patients did in terms of five of the subscales (Figure 3C). Both groups presented significant symptom improvement during the postoperative follow‐up period compared with the preoperative period (*p* < 0.05); however, the CNA group showed greater improvement than the control group did in terms of dizziness (Figure 3D–F).

## 4 Discussion

### 4.1 Major Findings

The main findings of this study are as follows: (1) Simple right atrial Ao‐SVC GP ablation could significantly improve patients’ basal and minimum HR, WCL, cSNRT, and heart rate variability. (2) The patients in the CNA group experienced less dizziness, improved activity tolerance, fewer episodes of recurrent syncope, and better overall QoL. CNA had no significant effect on the recurrence of PVCs or PSVT. (3) Ao‐SVC GP ablation did not significantly prolong the procedure time and was less invasive and safer. During the perioperative period and the average follow‐up period, no serious complications related to GP ablation occurred.

### 4.2 The Rationale for Right Atrial Ganglionated Plexus Ablation

Pachon et al. [25] performed the first CNA procedure for symptomatic bradycardia in 2005 and achieved favorable clinical results. Extensive knowledge of the intrinsic nervous system of the heart and the distribution of the GP has led to the recognition of CNA as a safe and effective therapy for patients without a well‐known indication for pacemaker implantation and for those who refuse pacemaker implantation [6, 7, 8, 10, 11, 12]. CNA primarily targets GP distributed over the atria, and in previous reports, GP ablation has been primarily performed in the LA, although approximately 50% of GP have been observed on the surface of the RA [14, 26]. Armour et al. [14] performed an anatomical distribution study of human atrial GPs and reported that a large number of GPs are distributed in the RA. In addition, the superior right atrial GP was described as being located on the posterior surface of the RA near the superior vena cava junction between the superior vena cava and the aorta, and this posterior region, also known as the Ao‐SVC GP or the “third fat pad,” is the point of connection for vagal inputs to the GP before innervation of the atria. As the first point of vagal afferents to the heart, most of the vagal fibers innervating the atria first pass through the Ao‐SVC GP and then extend over the other atrial GP and the atrial surface to innervate the sinoatrial node and AV node [16, 27]. Anatomical specificity determines the modulatory function of the Ao‐SVC GP for cardiac vagal tone.

This conclusion was also reflected in previous studies. Rebecchi et al. [15] reported the clinical data of two patients who experienced frequent syncopal episodes during a head‐up tilt test (HUTT) and a significant cardioinhibitory response. RFCA at the anatomical sites of the RA GP effectively prevented syncopal episodes and was considered a feasible method for prolonging the time to symptom onset and delaying pacemaker implantation. However, a 17‐year‐old male patient with VVS and recurrent syncope despite medication treatment, tilt training, and education on syncope prevention underwent endocardial ablation of right atrial Ao‐SVC GP via anatomical localization by Suenaga et al. [16] After 1 year of follow‐up, the patient's heart rate increased from 40 beats/min to 76 beats/min with no syncope during the follow‐up period, suggesting that Ao‐SVC GP ablation alone yields favorable results. Similarly, Aksu et al. [28] demonstrated that in patients with functional AV block, endocardial ablation by RA for selective vagal denervation seems reasonable. A related study by QIN et al. [7] demonstrated that Ao‐SVC GP ablation after left atrial GP ablation resulted in an additional increase in HR. This phenomenon could not be observed when other GPs were ablated, suggesting that Ao‐SVC GP may further modulate sinus node function. In addition, 18 patients with VVS were included in a recent prospective study of RFCA alone for GP in the RA using an anatomical approach, and after a mean follow‐up of 34.1 ± 6.1 months, syncope and recurrent prodromal symptoms were significantly relieved in the overall population and in symptomatic patients after ablation, suggesting that endomyocardial ablation of GP in the RA can be considered a safe and effective treatment for VVS [17].

Currently, there is no unified standard for GP ablation pathways and ablation strategy selection. In clinical studies related to GP ablation, different authors have reported the clinical effects of dual atrial ablation [25, 29, 30, 31], left atrial ablation alone [32, 33], and right atrial ablation alone [15, 16] on patients’ heart rate and symptom improvement. Most research centers adopt a dual atrial ablation strategy or a left atrial ablation strategy. In comparison, there are relatively few studies on right atrial GP ablation, and most of these are case reports, with relatively insufficient evidence of efficacy. A recent meta‐analysis [9] on the efficacy of GP ablation included 14 clinical studies that were categorized into three groups based on their intervention methods: isolated right atrial GP ablation (two studies), isolated left atrial GP ablation (five studies), and combined bilateral atrial GP ablation (seven studies). The results showed that all three ablation strategies significantly improved patients’ syncope symptoms. Compared with the other two strategies, isolated right atrial GP ablation had a higher syncope recurrence rate but a lower incidence of adverse events.

Several studies have demonstrated that ablation of right atrial GP improves AV conduction function and vagal tone. However, to our knowledge, no clinical studies have explored the long‐term efficacy and prognosis of ablation of right atrial Ao‐SVC GP for patients with vagal bradycardia. Thus, we designed the methodology of this study based on the anatomical distribution of atrial GP described in human studies. For patients with vagal bradycardia with PVCs or PSVT, we chose RFCA for paroxysmal tachyarrhythmia followed by anatomical localization of the right atrium for Ao‐SVC GP ablation to improve vagal bradycardia. Compared with biatrial GP ablation, right atrial GP ablation is easier to perform, with no need for septal puncture or left atrial modeling and mapping, no need for intraoperative analgesic and sedative drugs, and no need for postoperative anticoagulant drugs, which results in decreased surgical risk, surgery time, and surgical costs. Compared with anatomical localization in all other ablation procedures involving the right atrium, anatomical localization of Ao‐SVC GP is easy and is conducted far from the normal conduction bundle branches of the heart, which greatly reduces the risks of AV block, ablation injury, and prolonged surgery.

### 4.3 Selection of the GP Ablation Population

There are still no standardized criteria for the selection of populations and endpoints for GP ablation, and the optimal method to identify people with abnormally increased vagal tone is not completely clear. In addition to HRV, which is commonly used as an objective measure of autonomic tone, heart rate DC is a noninvasive and direct assessment method [18]. Zheng L et al. [34] reported that DC values decreased rapidly 1 day after GP ablation in patients without recurrent syncope and remained below baseline levels during the first postoperative year, suggesting that CNA may prevent recurrence by reducing vagal tone. Zheng L et al. [19] subsequently reported that DC was significantly greater in the syncope group than in the control group after comparing the relevant indices between the VVS group and the control group and noting that a DC > 7.5 ms was sufficient for monitoring cardiac vagal activity. Therefore, a heart rate DC > 7.5 ms was used as a quantitative criterion for diagnosing high vagal tone, while atropine positivity or cSNRT > 525 ms was used as an indicator for the diagnosis of SA node dysfunction and exclusion criteria to identify the most likely beneficiaries.

### 4.4 Clinical Outcomes and Safety of CNA

Improvements in sinus node function and HR were also demonstrated by Ao‐SVC GP modification in this study. The increase in HR immediately after Ao‐SVC GP ablation was significant compared with that before the procedure in 48 patients in the CNA group, accounting for 87.3% of the trial group population. After a mean follow‐up of 12.82 ± 6.67 months, the mean and minimum HR in the CNA group remained higher than those before the procedure (*p* < 0.05). The right atrial GP modification significantly improved the patient's WCL, cSNRT, HF and heart rate DC, suggesting that sinus node function and autonomic interneuron tone were modulated, which is consistent with the results of previous case reports [16]. However, we did not find a significant difference in LF/HF between the two groups, which may be related to late cardiac autonomic regeneration and recovery. In addition, no significant difference in the recurrence rate of PVCs or PSVT was observed between the two groups, suggesting that right atrial GP ablation had no significant effect on the recurrence rate of PVCs or PSVT. Although the symptoms and quality of life of patients in both groups improved compared with those in the preoperative period, the postoperative follow‐up results revealed that patients in the CNA group experienced more significant relief of dizziness, and the incidence of syncope in patients in the CNA group compared with that in the control group was significantly lower in the postoperative period than in the preoperative period (*p* = 0.032 < 0.05). In terms of QoL assessment, the scores of 7 of the 8 subscales of the SF‐36 significantly improved in the CNA group, with five subscales showing a greater improvement in scores compared with those in the control group (*p* < 0.05).

The results of this study show that combined right atrial Ao‐SVC GP ablation has a favorable effect on symptoms and quality of life in the study population, and the patient benefit is more significant. Unlike biatrial GP modification for vasovagal syncope, it appears that right‐atrial Ao‐SVC GP modification only for this population also yields good clinical results. The safety of single right atrial Ao‐SVC GP ablation was also confirmed in this study. Only one patient in the CNA group developed sinus tachycardia and was treated with metoprolol during the follow‐up period, and 1 patient in the CNA group and the control group suffered a complication associated with vascular puncture (pseudoaneurysm) and exhibited improvement after treatment, except for the patients who did not develop Ao‐SVC GP ablation‐related arrhythmias or other complications.

### 4.5 Clinical Implications

To our knowledge, this is the first clinical study to explore the long‐term efficacy and prognosis of single right atrial Ao‐SVC GP ablation for symptomatic bradycardia and the first to propose a one‐stop RFCA for vagal bradycardia and paroxysmal tachyarrhythmias. It not only improves patients’ heart rate and clinical symptoms and reduces the incidence of vasovagal syncope but also avoids pacemaker implantation and unnecessary nonphysiologic pacing.

### 4.6 Study Limitations

This study has several limitations. First, this was a single‐center study with a small sample size, and large‐scale randomized controlled trials are essential to further evaluate the effectiveness and safety of this new treatment. Second, the optimal method for identifying the population with vagal bradycardia without sinus node dysfunction and the GP ablation endpoint is still not completely clear. Moreover, the influence of cardiac autonomic nerves on the onset, maintenance, and termination of ventricular and supraventricular arrhythmias has been the subject of an unexhausted discussion, and the two may interact with each other. In this study, we observed the patients for 20 min after the end of the ablation of PVCs or PSVT. We again measured WCL and RR intervals, and we chose the exact target population as the test subjects. Nonetheless, the link between autonomic and paroxysmal tachyarrhythmia is difficult to rule out completely, and the connection needs to be explored further. Finally, although this study showed that Ao‐SVC GP ablation was effective in increasing the sinus rate and improving symptoms during the mean follow‐up period, previous studies have demonstrated the restoration of autonomic innervation, and longer‐term follow‐up is necessary to prove the efficacy of this approach.

## 5 Conclusion

Our research revealed that the single right atrial simplified Ao‐SVC GP ablation approach is safe and effective for treating vagal bradycardia. For vagal bradycardia with paroxysmal tachyarrhythmia, a consolidated surgical approach combining radiofrequency ablation for tachyarrhythmia with Ao‐SVC GP ablation for bradyarrhythmia is an innovative, safe, and effective treatment.

## Author Contributions

**Xinai Meng:** data curation, formal analysis, writing – original draft. **Senlin Huang:** investigation, writing – original draft. **Liwei He:** resources, visualization. **Tian Liu:** methodology, investigation. **Yanjia Chen:** project administration, writing – review and editing. **Huahua Li:** methodology, investigation. **Xingfu Huang:** funding acquisition, project administration, supervision, writing – review and editing.

## Conflicts of Interest

The authors declare no conflicts of interest.

## Data Availability

The data underlying this article will be shared on reasonable request to the corresponding author.
